# Evaluation of Extra-Prostatic Extension on Deep Learning-Reconstructed High-Resolution Thin-Slice T2-Weighted Images in Patients with Prostate Cancer

**DOI:** 10.3390/cancers16020413

**Published:** 2024-01-18

**Authors:** Mingyu Kim, Seung Ho Kim, Sujin Hong, Yeon Jung Kim, Hye Ri Kim, Joo Yeon Kim

**Affiliations:** 1 Department of Radiology, Haeundae Paik Hospital, Inje University College of Medicine, Busan 48108, Republic of Korea; 2 Department of Pathology, Haeundae Paik Hospital, Inje University College of Medicine, Busan 48108, Republic of Korea

**Keywords:** magnetic resonance imaging (MRI), deep learning, image reconstruction, prostate cancer

## Abstract

**Simple Summary:**

Extra-prostatic extension (EPE) is a well-known poor prognostic factor of prostate cancer. As such, accurate radiological diagnosis of EPE is important for urology surgeons due to its surgical implications prior to radical prostatectomy. T2-weighted imaging (T2WI) is a key sequence in prostate MRI for evaluating EPE. Recent technical advancements have allowed for the acquisition of thin-slice T2WI with resultant increased image noise. Deep learning reconstruction (DLR) techniques have been used to decrease the image noise in MRI; however, previous studies have focused mainly on image quality or acquisition times, and the efficacy of DLR regarding the diagnostic performance for EPE has not yet been reported. Our observations reveal that conventional 3 mm T2WI was better than 2 mm thin-slice T2WI with DLR with respect to diagnostic performance for EPE and image quality, thus supporting the minimal technical requirements described in the prostate imaging quality guidelines.

**Abstract:**

The aim of this study was to compare diagnostic performance for extra-prostatic extension (EPE) and image quality among three image datasets: conventional T2-weighted images (T2WI_conv_, slice thickness, 3 mm) and high-resolution thin-slice T2WI (T2WI_HR_, 2 mm), with and without deep learning reconstruction (DLR) in patients with prostatic cancer (PCa). A total of 88 consecutive patients (28 EPE-positive and 60 negative) diagnosed with PCa via radical prostatectomy who had undergone 3T-MRI were included. Two independent reviewers performed a crossover review in three sessions, in which each reviewer recorded five-point confidence scores for the presence of EPE and image quality using a five-point Likert scale. Pathologic topographic maps served as the reference standard. For both reviewers, T2WI_conv_ showed better diagnostic performance than T2WI_HR_ with and without DLR (AUCs, in order, for reviewer 1, 0.883, 0.806, and 0.772, *p* = 0.0006; for reviewer 2, 0.803, 0.762, and 0.745, *p* = 0.022). The image quality was also the best in T2WI_conv_, followed by T2WI_HR with DLR_ and T2WI_HR without DLR_ for both reviewers (median, in order, 3, 4, and 5, *p* < 0.0001). In conclusion, T2WI_conv_ was optimal in regard to image quality and diagnostic performance for the evaluation of EPE in patients with PCa.

## 1. Introduction

Multi-parametric MRI of the prostate gland is a cornerstone in the diagnosis and evaluation of prostate cancer (PCa). Although technical requirements have been given in the second version of Prostate Imaging-Reporting and Data System (PI-RADS v.2.1), the prostate MRI parameters of acquisition and scan quality vary between different institutions [1,2,3]. However, as the detection of PCa is impaired by poor image quality and artifacts in the acquired images, methods for image optimization have been studied [4,5]. To facilitate the improvement in the image quality of prostate MRI, the Prostate Imaging Quality (PI-QUAL) score has been introduced, including minimal technical requirements described in the PI-RADS, as well as several criteria for visual assessment [3,6].

T2-weighted imaging (T2WI) is a key sequence in multi-parametric MRI, which is important in the evaluation of the zonal anatomy of the prostate gland and neighboring structures, extra-prostatic extension (EPE), seminal vesical and neurovascular bundle invasion, as well as playing a role as the dominant sequence for the evaluation of transitional zone (TZ) malignancies [7]. Of these, EPE is a well-known poor prognostic factor, and so the accurate radiological diagnosis of EPE is important for urology surgeons due to its surgical implications for surgical planning prior to radical prostatectomy [8,9].

The minimal technical requirements of PI-QUAL for T2WI are ≤0.4 mm in the frequency-encoding direction, ≤0.7 mm in the phase-encoding direction for in-plane resolution, and 3 mm for slice thickness (ST) [6]. Recent technical advancements in MRI have allowed for the acquisition of three-dimensional (3D) T2WI with resultant thinner STs. Previous studies have reported the comparable performance of 3D-T2WI to conventional T2WI regarding the diagnosis and characterization of prostate cancer and evaluation of EPE [10,11,12]. A potential drawback of thin-slice T2WI is a decrease in the signal-to-noise ratio (SNR) and an increase in motion-related artifacts in images [7,13]. Deep learning reconstruction (DLR) techniques have been used to decrease image artifacts and noise, and previous studies have applied DLR to cardiac, musculoskeletal, and neuroimaging, revealing promising results in terms of improved image quality [14,15,16,17,18]. Previous studies on DLR in prostate MRI have mainly focused on its effect on image quality or acquisition time, and comparable or improved image quality with DLR has been observed [19,20,21]. However, to the best of our knowledge, the effect of DLR on the diagnostic performance for the evaluation of EPE in PCa has been scarcely reported [22]. Therefore, we aimed to compare the diagnostic performance of three image datasets—conventional T2WI (T2WI_conv_) and high-resolution thin-slice T2WI (T2WI_HR_) with and without DLR—in order to find the optimal image set for the evaluation of EPE in patients with PCa.

## 2. Materials and Methods

The institutional review board approved this retrospective study, and the requirement for informed consent was waived.

### 2.1. Patient Selection Criteria

**Figure 1:**
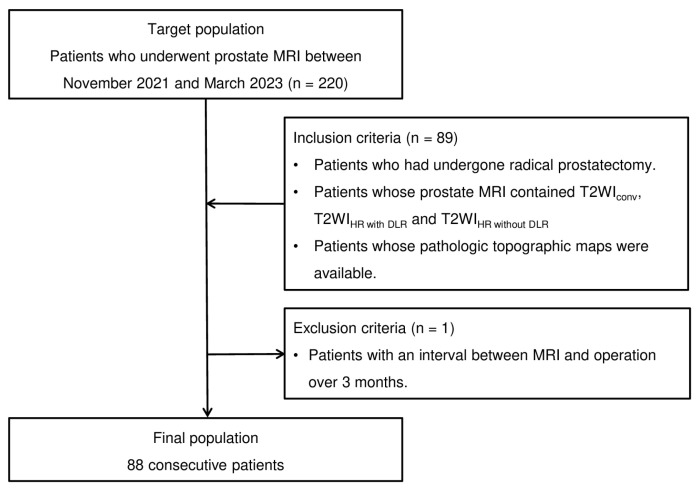
Flowchart of the case-accrual process.

A total of 220 patients who underwent prostate MRI between November 2021 and March 2023 were initially considered eligible for this study. Among them, 88 patients satisfying the following inclusion criteria were finally included in the study. The inclusion criteria were as follows: (1) patients who had undergone radical prostatectomy; (2) patients whose prostate MRI scans contained three image datasets; and (3) patients who had a pathologic topographic map (whole-mount slide) and relevant information such as Gleason scores. The patient enrollment process is summarized in Figure 1.

### 2.2. Magnetic Resonance Imaging

All MRI examinations were performed using a 3.0-T MR scanner (Signa Architect, GE HealthCare, Chicago, IL, USA) with a body coil (AIR^TM^ Anterior Array, GE HealthCare Coils, Aurora, OH, USA). The scanning protocol consisted of the following sequences: (1) axial, coronal, and sagittal T2WI_conv_; (2) axial T2WI_HR_; (3) axial diffusion-weighted imaging (DWI) sequences (b-values of 0 and 1000 s/mm^2^); and (4) apparent diffusion coefficient (ADC) maps generated from b-values of 0 and 1000 s/mm^2^. The voxel sizes of T2WI_conv_ and T2WI_HR_ were 0.5 × 0.7 × 3 mm^3^ and 0.5 × 0.5 × 2 mm^3^, respectively. The MRI parameters are detailed in Table 1. The DICOM images of all sequences were uploaded and reviewed from a picture archiving and communication system (PACS) workstation (M6; INFINITT Healthcare, Seoul, Republic of Korea).

### 2.3. Deep Learning Reconstruction of T2WI_HR_

A commercially available DLR algorithm (AIR^TM^ Recon DL, GE HealthCare) was applied to T2WI_HR_ to create T2WI_HR with DLR_. The DLR algorithm employed in this study incorporates a deep convoluted neural network to produce images that are consistent with the original image data while reducing Gibbs ringing artifacts, sharpness of object edges, and image noise, thus improving the SNR ratio [18]. The level of de-noising achieved by the DLR algorithm can be selected from three presets: low, medium, or high strength. A medium strength was chosen for this study. The medium strength achieves a 50% reduction in image noise, compared with the anticipated image noise, whereas the low strength yields a 30% reduction and the high strength yields a 75% reduction [18].

### 2.4. Image Analysis

The three image sets (axial T2WI_conv_, T2WI_HR with DLR_, and T2WI_HR without DLR_) were reviewed in three separate sessions with a cross-over review design. The images for review in each session comprised approximately one-third of each image set, and the order of images for review was randomized. The images were independently reviewed by two radiologists (with 4 and 3 years of experience in interpreting prostate MRI, respectively). The three review sessions were separated by a 4-week interval, in order to reduce recall bias. Coronal and sagittal T2WI_conv_, axial DWI, and corresponding ADC maps (b2000) of each patient were also provided in each review session. Reviewers were blinded to the clinical and pathologic data of the patients, except for the fact that all patients had pathologically proven PCa. Reviewers identified the largest tumor (i.e., the index tumor) in the prostate gland and manually drew the tumor in a schematic diagram of the prostate gland with regard to the level and zonal anatomy. The reviewers then assessed the presence of EPE in the index tumor and assigned a confidence score for EPE based on a 5-point scale describing their confidence in the finding (1 = definitely absent, 2 = probably absent, 3 = equivocal, 4 = probably present, and 5 = definitely present). The window width and level values could be adjusted by each reviewer.

### 2.5. Image Quality

Contemporaneously to confidence scoring for EPE, the two reviewers also subjectively assessed the image quality of each image dataset. A subjective image quality score was assigned to each image set based on a 5-point Likert scale, where the score represented the number of the visual assessment criteria that the images satisfied. For example, if an image set satisfied only four out of five criteria, a subjective image quality score of 4 would be assigned. The visual assessment criteria were partially based on those described in PI-QUAL [6] and were as follows: (a) prostatic capsule is clearly delineated; (b) seminal vesicles are clearly delineated; (c) neurovascular bundles are clearly delineated; (d) absence of noise; and (e) absence of artifacts such as motion artifacts.

For quantitative analysis of image quality, the SNR and contrast-to-noise ratio (CNR) were assessed for the three image datasets by a third radiologist. The SNR for each image dataset was defined as the mean signal intensity (SI) value of the non-cancerous prostate parenchyma divided by the image noise, while the CNR for each image data set was defined as the absolute SI value of the index tumor (defined as the difference between the mean SI value of the index tumor and the mean SI value of the non-cancerous prostate parenchyma) divided by the image noise. Image noise was defined as the standard deviation of the SI value of the skeletal muscle of the pelvic wall.

### 2.6. Reference Standard

All patients underwent radical prostatectomy. The resected specimens were evaluated with respect to EPE and the Gleason grading system [23] by a dedicated pathologist. Whole-mount histopathology served as the reference standard for the ground truth of the presence of EPE, as well as tumor location and extent. A senior radiologist with 14 years of experience in interpreting prostate MRI determined whether the pathologic index tumor coincided with the index tumor identified by the two reviewers in each review session.

### 2.7. Statistical Analysis

The MedCalc software for Windows (MedCalc Software version 19.6.1, Mariakerke, Belgium) was used for the statistical analyses. Statistical significance was set at *p* < 0.05. The detection rate (i.e., sensitivity) with regard to the index tumor was compared using McNemar’s test. Receiver operating characteristic (ROC) curve analyses were performed for the diagnostic performance of each reviewer for a specific dataset. The area under the ROC curve (AUC) was calculated and compared pairwise to compare diagnostic performances between any two image datasets. For each reviewer, the image quality of each dataset was compared using the Wilcoxon signed-rank test. For the assessment of quantitative image quality, SNR and CNR were compared through repeated measures ANOVA.

## 3. Results

### 3.1. Patient Demographics

The study population comprised 28 patients with EPE and 60 patients without EPE. The mean interval from pre-operative prostate MRI to radical prostatectomy was 33.5 days (range: 1–88 days), and the mean prostate-specific antigen (PSA) level was 20.37 ng/dL (range: 0.85–154 ng/dL). The number of patients with each Gleason score (GS) was as follows: GS6 (n = 25), GS7 (3 + 4, n = 37; 4 + 3, n = 18), GS8 (n = 3), and GS9 (n = 5). The number of patients with each pathologic T-stage was as follows: T2 (n = 60), T3a (n = 13), and T3b (n = 15). The specific demographic data are presented in Table 2.

### 3.2. Comparison of Detection Rates for the Index Tumor

For reviewer 1, the detection rate of the index tumor was 85% for all three image datasets. For reviewer 2, the detection rate of the index tumor on T2WI_conv_, T2WI_HR with DLR_, and T2WI_HR without DLR_ was 87%, 86%, and 86%, respectively. The detection rates did not present significant differences among the three image datasets for both reviewers (*p* > 0.05).

### 3.3. Comparison of Diagnostic Performance for EPE

**Figure 2:**
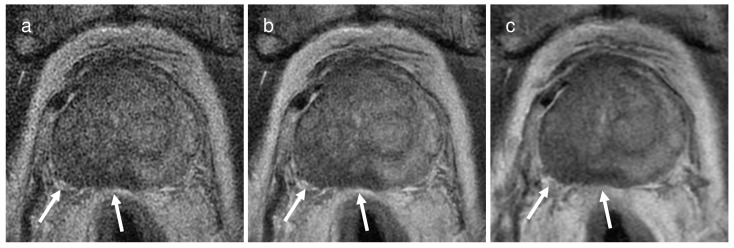
A representative case of a 74-year-old man with a Gleason score of 9 in the right middle peripheral zone demonstrating extra-prostatic extension through the right posterolateral capsule (arrows) on (**a**) a 2 mm thin-slice T2WI without deep learning reconstruction (DLR), (**b**) the same 2 mm T2WI with DLR, and (**c**) conventional T2WI with 3 mm slice thickness. For both reviewers, the confidence score for presence of extra-prostatic extension increased from 4 (probably present) on the 2 mm thin-slice T2WI with or without DLR to 5 (definitely present) on conventional T2WI.

For reviewer 1, T2WI_conv_ (AUC, 0.883; 95% confidence interval (CI), 0.784–0.947) showed a significantly better diagnostic performance compared to T2WI_HR with DLR_ (AUC, 0.806; 95% CI, 0.694–0.891; *p =* 0.0057) and T2WI_HR without DLR_ (AUC, 0.772; 95% CI, 0.656–0.864; *p =* 0.0006). For reviewer 2, T2WI_conv_ (AUC, 0.803; 95% CI, 0.691–0.888) also showed a significantly greater AUC compared to T2WI_HR with DLR_ (AUC, 0.762; 95% CI, 0.646–0.855; *p* = 0.0220) and T2WI_HR without DLR_ (AUC, 0.745; 95% CI, 0.627–0.841; *p* = 0.0277). For both reviewers, the diagnostic performance between T2WI_HR with DLR_ and T2WI_HR without DLR_ did not present a significant difference (for reviewer 1, *p* = 0.1610; for reviewer 2, *p* = 0.3175). Representative images are presented in Figure 2, and the diagnostic performance is summarized in Table 3.

### 3.4. Comparison of Image Quality

#### 3.4.1. Qualitative Analysis

**Figure 3:**
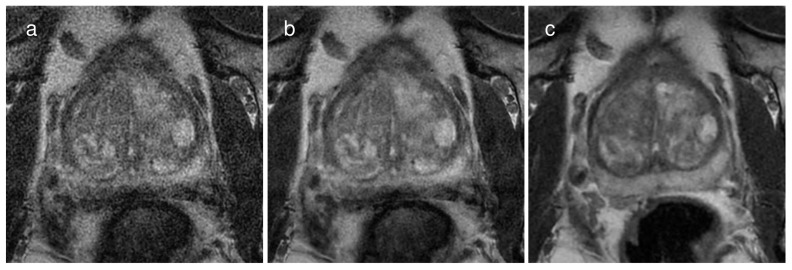
A representative case of a 67-year-old man with a Gleason score of 6 demonstrating the image quality of each image dataset on (**a**) a 2 mm thin-slice T2WI without deep learning reconstruction (DLR), (**b**) the same T2WI with DLR, and (**c**) conventional T2WI with 3 mm slice thickness. The subjective image quality score was the best on conventional T2WI, followed by the thin-slice T2WI with DLR and the same T2WI without DLR. Specifically, the scores were 5, 4, and 3, respectively, for reviewer 1 and 4, 3, and 2, respectively, for reviewer 2.

For reviewer 1, the subjective image quality score of T2WI_conv_ (median, 5; interquartile range, 5–5) was greater than T2WI_HR with DLR_ (median, 4; interquartile range, 3–5; *p* < 0.0001) and T2WI_HR without DLR_ (median, 3; interquartile range, 3–4; *p* < 0.0001). For reviewer 2, the subjective image quality score of T2WI_conv_ (median, 5; interquartile range, 4–5) was also greater than T2WI_HR with DLR_ (median, 4; interquartile range, 3–4; *p* < 0.0001) and T2WI_HR without DLR_ (median, 3; interquartile range, 3–4; *p* < 0.0001)**.** For both reviewers, T2WI_HR with DLR_ had a significantly higher subjective image quality score compared to T2WI_HR without DLR_ (*p* < 0.0001). Representative images are presented in Figure 3.

#### 3.4.2. Quantitative Analysis

The SNR of T2WI_conv_ (mean ± SD, 22.17 ± 7.02) was the highest, followed by T2WI_HR with DLR_ (15.81 ± 4.80) and T2WI_HR without DLR_ (8.71 ± 2.24) (*p* < 0.001). Similarly, the CNR of T2WI_conv_ (6.05 ± 4.16) was the highest, followed by T2WI_HR with DLR_ (4.41 ± 3.16) and T2WI_HR without DLR_ (2.33 ± 1.57) (*p* < 0.001). The measurement results are summarized in Table 4.

## 4. Discussion

The results of our study revealed that the diagnostic performance for EPE in PCa was greater in T2WI_conv_ compared to T2WI_HR_, while DLR did not significantly improve the diagnostic performance in T2WI_HR_. In terms of image quality, T2WI_conv_ demonstrated a higher image quality than T2WI_HR_ both subjectively and quantitatively, while DLR significantly improved the image quality of T2WI_HR_.

As PI-RADS v.2.1 recommends the T2WI as the key sequence for the detection of TZ cancer [1] and DWI for peripheral zone (PZ) cancer, we believe that the benefits of DLR in T2WI_HR_ could be expected in cases of TZ cancer. Our observation that DLR did not enhance the diagnostic performance in evaluating EPE can be explained by several factors. First, the proportion of EPE in TZ cancer should be considered. The proportion was approximately 20% (6/28), whereas that of PZ cancer was 32% (17/53). Second, the detection of TZ cancer using T2WI alone is very challenging; thus, supplementary reading of DWI might affect the diagnostic decision on EPE. The better performance of T2WI_conv_ could be attributable to the better SNR and CNR compared to T2WI_HR_ combined (i.e., whether DLR or not). Out of the 28 patients with EPE in this study, 17 patients had PZ cancer (60%) and the EPE was predominant when the PZ cancer invaded the posterolateral capsule. Therefore, this posterolateral area was the main site for the radiologists to confidently evaluate EPE using the three image datasets. Consequently, in our opinion, T2WI_conv_ benefited from the better conspicuity in anatomical structures resulting from the improved SNR and CNR of T2WI_conv_, compared to the other image datasets. Our observations support the PI-QUAL requirement for image acquisition parameters. A 2 mm ST, even with the aid of DLR, did not outperform the conventional 3 mm on T2WI, which is the PI-QUAL guideline requirement for image acquisition.

To the best of our knowledge, the effect of DLR on the detection of EPE has not been previously reported with regard to enhanced spatial resolution. Beyond the spatial resolution, Park et al. [22] compared conventional T2WI (ST, 3 mm) and T2WI with a shorter acquisition time (i.e., fast T2WI) with and without DLR in terms of diagnostic performance for the diagnosis of EPE in terms of enhanced temporal resolution. They achieved an enhanced temporal resolution from 4 min to 2 min 30 s by adopting a reduced repetition time (from 4000 to 2800 ms) and number of excitations (from 1.5 to 1.0), and they employed the same commercially available DLR algorithm (AIR^TM^ Recon DL). Their results revealed that T2WI_conv_ showed a comparable diagnostic performance to fast T2WI with DLR for all three reviewers and a mixed response to applying DLR to fast T2WI. Specifically, an improved performance was observed for two reviewers (AUCs, for reviewer 1, T2WI_conv_, fast T2WI _with DLR_, fast T2WI _without DLR_ in order, 0.85, 0.86, 0.75; for reviewer 2, 0.82, 0.82, 0.73, respectively), whereas stationary performance was revealed in one reviewer (AUCs, 0.79, 0.81, 0.74, respectively). Our results partially correspond with the above study, in that T2WI_conv_ presented the best performance and DLR did not improve the diagnostic performance for diagnosis of EPE.

The effect of DLR on the image quality of prostate MRI has been more frequently studied. Wang et al. [19] compared four image datasets—T2WI (ST, 3 mm) with the endo-rectal coil signal turned on and off, both with and without DLR—in terms of image quality and visualization of various anatomic structures and the tumor. They also employed the same commercial DLR software (AIR^TM^ Recon DL). The image quality of T2WI with DLR when the endo-rectal coil signal was turned off was significantly higher than the other three image sets (*p* < 0.01). This sequence was also most frequently chosen as the best one for the visualization of major anatomical landmarks and the tumor. Park et al. [22] also reported a significant improvement in the SNR (fast T2WI _with DLR_ vs. fast T2WI _without DLR_, 14.7 vs. 8.8, *p* < 0.001), CNR (6.5 vs. 3.4, *p* < 0.001), and lesion delineation quality by subjective assessment and subjective image quality scores (from 1 = non-diagnostic to 5 = excellent; all readers, 4.0 vs. 3.0; all *p* < 0.001) when DLR was applied to fast MRI. Our study corresponds well with the above studies, in that the subjective image quality as well as quantitative SNR and CNR could be improved with DLR [19,22].

Beyond the two-dimensional (2D) acquisition of T2WI, the benefits of 3D-T2WI in prostate MRI include reduced acquisition time and post-processing in multiple planes. However, the effect on the diagnostic performance for evaluating EPE is a subject with more conflicting data [13]. Choi et al. [10] reported no significant difference between conventional T2WI (ST, 3 mm) and 3D-T2WI (0.6 mm) in terms of the sensitivity (for reader 1, 28% vs. 48%, *p* = 0.375; for reader 2, 64% vs. 60%, *p* = 1.000) and specificity (for reader 1, all 91%; for reader 2, all 76%) for detecting EPE. Caglic et al. [24] found that 3D-T2WI (1 mm) had a similar diagnostic performance to conventional 2D-T2WI (3 mm) (AUC, 0.877 vs. 0.835, *p* = 0.17; sensitivity, 75% vs. 65%, *p* = 0.058; and specificity, 84% vs. 86%, *p* = 0.705) in the detection of EPE. Rosenkrantz et al. [11] also compared 3D- (ST, 1 mm) and 2D-T2WI (ST, 3 mm) for the diagnosis of EPE and reported a similar accuracy (3D T2WI vs. 2D T2WI, 68% vs. 74%, *p* = 0.6250). At present, PI-RADS v.2.1 recommends an ST of 3 mm and, while 3D axial acquisitions may be used as a supplementary sequence, the altered tissue contrast should be treated with caution, and the possibility that in-plane resolution may be lower in 3D acquisitions also should be considered [1,3]. As the added value of 3D-T2WI in the diagnosis of EPE is a subject of debate, we believe that the current guidelines are sufficient for the diagnosis of EPE, and the acquisition of 3D-T2WI could be conducted in prudence when more robust evidence suggests it concordantly.

Emphasizing the pros and cons of DLR is worthy of notice because DLR has been increasingly applied to radiological imaging with the benefits of reducing acquisition times while preserving image quality, as well as improving image quality while maintaining acquisition times. In regard to shortening acquisition time, DLR has been applied to fast-spin echo imaging in prostate MRI, and in particular, a reduction in acquisition time of up to 65% has been shown in T2-weighted fast-spin echo imaging [25]. DLR has also been implemented for the purpose of improving image quality [19,22,26]. Although the application of DLR itself may be harmless to patients, potential cons may include the additional cost of DLR software and the observations that the improved image quality may not always lead to improved diagnostic performance for specific diagnostic tasks like ours. In our opinion, although diagnostic performance based on radiologists’ perceptions of minute anatomical structures or lesions may benefit from the improved image quality after the application of DLR to images with noise [27], diagnostic tasks based on the integration of various image findings may not enhance radiologists’ diagnostic performance in spite of improved image quality after applying DLR [22,28,29,30].

There were several limitations to our study. First, only patients who had undergone radical prostatectomy were included in our study. As advanced, recurrent, or metastatic PCa may undergo systemic therapy, rather than surgical therapy [31], a selection bias against more advanced cases might have been introduced; therefore, it is possible that advanced metastatic cases with EPE were excluded. However, such a criterion was necessary in order to obtain topographic maps with whole-mount pathology, thus providing a ground truth for the MRI findings to be compared with. Second, acquisition parameters other than the ST were slightly different for T2WI_HR_; thus, the in-plane resolution differed slightly due to different field-of-view and matrix sizes. Further studies are needed to elucidate the isolated effect of thinner STs in the evaluation of EPE. Third, DWI and ADC maps were provided to the reviewers. As DWI and ADC maps are crucial sequences for the detection of PCa, especially for PZ cancers [1,3], the detection rate may not represent the isolated effect of T2WI sequences. However, DWI and ADC sequences are a mainstay in multi-parametric MRI, with a well-established role in the diagnosis of PCa. Therefore, our study adopted daily clinical practice, in that T2WI was reviewed alongside DWI and ADC maps.

## 5. Conclusions

In conclusion, T2WI_conv_ with a 3 mm ST was optimal with respect to diagnostic performance as well as image quality for the evaluation of EPE in patients with PCa. Thin-slice T2WI with a 2 mm ST did not outperform T2WI_conv_, even with the aid of DLR. Our results support the minimal technical requirements described in the PI-QUAL guidelines, which recommend an ST of 3 mm in the acquisition of axial T2WI.

## Figures and Tables

**Table 1:** MRI sequence parameters.

Parameter	T2WI_conv_ (Axial, Sagittal, and Coronal)	T2WI_HR_	DWI (b-values of 0 and 1000 s/mm^2^)
TR	4680–4930	3240–3270	5170–5240
TE	75–100	75–85	88–89
ETL	15	13	2
Slice thickness	3.0 mm	2.0 mm	3.0 mm
Slice gap	0.3 mm	0.0 mm	0.3 mm
Matrix size (axial)	400 × 320	320 × 320	120 × 120
NEX	1	1	b0, 2 b1000, 4
FOV (mm)	220 × 220	160 × 160	240 × 240
Acquisition time	1 min 28 s–1 min 51 s	4 min 28 s	2 min 32 s

Conventional T2-weighted images (T2WI_conv_); High-resolution thin-slice T2-weighted images (T2WI_HR_); Repetition time (TR); Echo time (TE); Echo train length (ETL); Field of view (FOV); Number of excitations (NEX); Diffusion-weighted imaging (DWI).

**Table 2:** Demographic data of the study population.

Parameter	Study Population (n = 88)
Mean age, years (range)	70.86 (52–86)
Mean PSA, ng/mL (range)	20.37 (0.85–154)
Mean interval from MRI to radical prostatectomy, days (range)	33.53 (1–88)
Presence of EPE, n (%)	28 (32%)
Pathologic T stage-n (%)	
T2	60 (68)
T3a	13 (15)
T3b	15 (17)
Gleason score, n (%)	
6	25 (28)
7	55 (63)
3 + 4	37
4 + 3	18
8	3 (3)
9	5 (6)
Tumor location, n (%)	
Peripheral zone	53 (60)
Transitional zone	28 (32)
Anterior fibromuscular stroma	2 (2)

Prostate-specific antigen (PSA); Extra-prostatic extension (EPE).

**Table 3:** Comparison of diagnostic performance and predictive values of T2WI_conv_, T2WI_HR with DLR_, and T2WI_HR without DLR_.

Image Sets	Reviewer 1							Reviewer 2						
	AUC	Sensitivity	Specificity	PPV	NPV	Accuracy	*p* Value	AUC	Sensitivity	Specificity	PPV	NPV	Accuracy	*p* Value
T2WI_conv_	0.883	77	96	91	89	89	0.0057 *	0.803	63	96	89	82	84	0.0220 *
T2WI_HR with DLR_	0.806	62	86	70	81	77	0.0006 ^†^	0.762	59	90	76	80	79	0.0277 ^†^
T2WI_HR without DLR_	0.772	46	89	69	76	74	0.1610 ^‡^	0.745	44	89	69	75	73	0.3175 ^‡^

* T2WI_conv_ vs. T2WI_HR with DLR_. ^†^ T2WI_conv_ vs. T2WI_HR without DLR_. ^‡^ T2WI_HR with DLR_ vs. T2WI_HR without DLR._ Conventional T2-weighted images (T2WI_conv_); High-resolution thin-slice T2-weighted images with deep learning reconstruction (T2WI_HR with DLR_); High-resolution thin-slice T2-weighted images without deep learning reconstruction (T2WI_HR without DLR_); Area under the receiver operating characteristic curve (AUC); Positive predictive value (PPV); Negative predictive value (NPV).

**Table 4:** Quantitative image quality analysis of T2WI_conv_, T2WI_HR with DLR_, and T2WI_HR without DLR_.

Image Set	Signal-to-Noise Ratio	*p* Value	Contrast-to-Noise Ratio	*p* Value
T2WI_conv_	22.17 ± 7.02	<0.001 *	6.05 ± 4.16	<0.001 *
T2WI_HR with DLR_	15.81 ± 4.80	<0.001 ^†^	4.41 ± 3.16	<0.001 ^†^
T2WI_HR without DLR_	8.71 ± 2.24	<0.001 ^‡^	2.33 ± 1.57	<0.001 ^‡^

Data are presented in mean ± standard deviations. * T2WI_conv_ vs. T2WI_HR with DLR_. ^†^ T2WI_HR with DLR_ vs. T2WI_HR without DLR_. ^‡^ T2WI_conv_ vs. T2WI_HR without DLR_. Conventional T2-weighted images (T2WI_conv_); High-resolution thin-slice T2-weighted images with deep learning reconstruction (T2WI_HR with DLR_); High-resolution thin-slice T2-weighted images without deep learning reconstruction (T2WI_HR without DLR_).

## Data Availability

The data presented in this study are available on request from the corresponding author. The data are not publicly available due to patients’ privacy.

## References

[1] Turkbey B., Rosenkrantz A.B., Haider M.A., Padhani A.R., Villeirs G., Macura K.J., Tempany C.M., Choyke P.L., Cornud F., Margolis D.J. (2019). Prostate Imaging Reporting and Data System Version 2.1: 2019 Update of Prostate Imaging Reporting and Data System Version 2. Eur. Urol..

[2] Esses S.J., Taneja S.S., Rosenkrantz A.B. (2018). Imaging Facilities’ Adherence to PI-RADS v2 Minimum Technical Standards for the Performance of Prostate MRI. Acad. Radiol..

[3] Weinreb J.C., Barentsz J.O., Choyke P.L., Cornud F., Haider M.A., Macura K.J., Margolis D., Schnall M.D., Shtern F., Tempany C.M. (2016). PI-RADS Prostate Imaging—Reporting and Data System: 2015, Version 2. Eur. Urol..

[4] Caglic I., Barrett T. (2019). Optimising prostate mpMRI: Prepare for success. Clin. Radiol..

[5] Jambor I. (2017). Optimization of prostate MRI acquisition and post-processing protocol: A pictorial review with access to acquisition protocols. Acta Radiol. Open.

[6] Giganti F., Kirkham A., Kasivisvanathan V., Papoutsaki M.V., Punwani S., Emberton M., Moore C.M., Allen C. (2021). Understanding PI-QUAL for prostate MRI quality: A practical primer for radiologists. Insights Imaging.

[7] Gupta R.T., Spilseth B., Patel N., Brown A.F., Yu J. (2016). Multiparametric prostate MRI: Focus on T2-weighted imaging and role in staging of prostate cancer. Abdom. Radiol..

[8] Magi-Galluzzi C., Evans A.J., Delahunt B., Epstein J.I., Griffiths D.F., van der Kwast T.H., Montironi R., Wheeler T.M., Srigley J.R., Egevad L.L. (2011). International Society of Urological Pathology (ISUP) Consensus Conference on Handling and Staging of Radical Prostatectomy Specimens. Working group 3: Extraprostatic extension, lymphovascular invasion and locally advanced disease. Mod. Pathol..

[9] McClure T.D., Margolis D.J., Reiter R.E., Sayre J.W., Thomas M.A., Nagarajan R., Gulati M., Raman S.S. (2012). Use of MR imaging to determine preservation of the neurovascular bundles at robotic-assisted laparoscopic prostatectomy. Radiology.

[10] Choi M.H., Lee Y.J., Jung S.E., Han D. (2023). High-resolution 3D T2-weighted SPACE sequence with compressed sensing for the prostate gland: Diagnostic performance in comparison with conventional T2-weighted images. Abdom. Radiol..

[11] Rosenkrantz A.B., Neil J., Kong X., Melamed J., Babb J.S., Taneja S.S., Taouli B. (2010). Prostate cancer: Comparison of 3D T2-weighted with conventional 2D T2-weighted imaging for image quality and tumor detection. AJR Am. J. Roentgenol..

[12] Polanec S.H., Lazar M., Wengert G.J., Bickel H., Spick C., Susani M., Shariat S., Clauser P., Baltzer P.A.T. (2018). 3D T2-weighted imaging to shorten multiparametric prostate MRI protocols. Eur. Radiol..

[13] Lim K.K., Noe G., Hornsey E., Lim R.P. (2014). Clinical applications of 3D T2-weighted MRI in pelvic imaging. Abdom. Imaging.

[14] van der Velde N., Hassing H.C., Bakker B.J., Wielopolski P.A., Lebel R.M., Janich M.A., Kardys I., Budde R.P.J., Hirsch A. (2021). Improvement of late gadolinium enhancement image quality using a deep learning-based reconstruction algorithm and its influence on myocardial scar quantification. Eur. Radiol..

[15] Hahn S., Yi J., Lee H.J., Lee Y., Lim Y.J., Bang J.Y., Kim H., Lee J. (2022). Image Quality and Diagnostic Performance of Accelerated Shoulder MRI With Deep Learning-Based Reconstruction. AJR Am. J. Roentgenol..

[16] Lee D.H., Park J.E., Nam Y.K., Lee J., Kim S., Kim Y.H., Kim H.S. (2021). Deep learning-based thin-section MRI reconstruction improves tumour detection and delineation in pre- and post-treatment pituitary adenoma. Sci. Rep..

[17] Sun S., Tan E.T., Mintz D.N., Sahr M., Endo Y., Nguyen J., Lebel R.M., Carrino J.A., Sneag D.B. (2022). Evaluation of deep learning reconstructed high-resolution 3D lumbar spine MRI. Eur. Radiol..

[18] Lebel R.M. (2020). Performance characterization of a novel deep learning-based MR image reconstruction pipeline. arXiv.

[19] Wang X., Ma J., Bhosale P., Ibarra Rovira J.J., Qayyum A., Sun J., Bayram E., Szklaruk J. (2021). Novel deep learning-based noise reduction technique for prostate magnetic resonance imaging. Abdom. Radiol..

[20] Kim E.H., Choi M.H., Lee Y.J., Han D., Mostapha M., Nickel D. (2021). Deep learning-accelerated T2-weighted imaging of the prostate: Impact of further acceleration with lower spatial resolution on image quality. Eur. J. Radiol..

[21] Gassenmaier S., Warm V., Nickel D., Weiland E., Herrmann J., Almansour H., Wessling D., Afat S. (2023). Thin-Slice Prostate MRI Enabled by Deep Learning Image Reconstruction. Cancers.

[22] Park J.C., Park K.J., Park M.Y., Kim M.H., Kim J.K. (2022). Fast T2-Weighted Imaging With Deep Learning-Based Reconstruction: Evaluation of Image Quality and Diagnostic Performance in Patients Undergoing Radical Prostatectomy. J. Magn. Reson. Imaging.

[23] Epstein J.I., Egevad L., Amin M.B., Delahunt B., Srigley J.R., Humphrey P.A. (2016). The 2014 International Society of Urological Pathology (ISUP) Consensus Conference on Gleason Grading of Prostatic Carcinoma: Definition of Grading Patterns and Proposal for a New Grading System. Am. J. Surg. Pathol..

[24] Caglic I., Povalej Brzan P., Warren A.Y., Bratt O., Shah N., Barrett T. (2019). Defining the incremental value of 3D T2-weighted imaging in the assessment of prostate cancer extracapsular extension. Eur. Radiol..

[25] Gassenmaier S., Afat S., Nickel D., Mostapha M., Herrmann J., Othman A.E. (2021). Deep learning-accelerated T2-weighted imaging of the prostate: Reduction of acquisition time and improvement of image quality. Eur. J. Radiol..

[26] Gassenmaier S., Küstner T., Nickel D., Herrmann J., Hoffmann R., Almansour H., Afat S., Nikolaou K., Othman A.E. (2021). Deep Learning Applications in Magnetic Resonance Imaging: Has the Future Become Present?. Diagnostics.

[27] Kaniewska M., Deininger-Czermak E., Lohezic M., Ensle F., Guggenberger R. (2023). Deep Learning Convolutional Neural Network Reconstruction and Radial k-Space Acquisition MR Technique for Enhanced Detection of Retropatellar Cartilage Lesions of the Knee Joint. Diagnostics.

[28] Johnson P.M., Lin D.J., Zbontar J., Zitnick C.L., Sriram A., Muckley M., Babb J.S., Kline M., Ciavarra G., Alaia E. (2023). Deep Learning Reconstruction Enables Prospectively Accelerated Clinical Knee MRI. Radiology.

[29] Hahn S., Yi J., Lee H.J., Lee Y., Lee J., Wang X., Fung M. (2023). Comparison of deep learning-based reconstruction of PROPELLER Shoulder MRI with conventional reconstruction. Skelet. Radiol..

[30] Feuerriegel G.C., Weiss K., Kronthaler S., Leonhardt Y., Neumann J., Wurm M., Lenhart N.S., Makowski M.R., Schwaiger B.J., Woertler K. (2023). Evaluation of a deep learning-based reconstruction method for denoising and image enhancement of shoulder MRI in patients with shoulder pain. Eur. Radiol..

[31] Virgo K.S., Rumble R.B., de Wit R., Mendelson D.S., Smith T.J., Taplin M.E., Wade J.L., Bennett C.L., Scher H.I., Nguyen P.L. (2021). Initial Management of Noncastrate Advanced, Recurrent, or Metastatic Prostate Cancer: ASCO Guideline Update. J. Clin. Oncol..

